# Clinical outcome of biportal endoscopic revisional lumbar discectomy for recurrent lumbar disc herniation

**DOI:** 10.1186/s13018-020-02087-6

**Published:** 2020-11-23

**Authors:** Min-Seok Kang, Jin-Ho Hwang, Dae-Jung Choi, Hoon-Jae Chung, Jong-Hwa Lee, Hyong-Nyun Kim, Hyun-Jin Park

**Affiliations:** 1 Department of Orthopedic Surgery, Bumin Hospital, Seoul, Republic of Korea; 2 Department of Orthopedic Surgery, Himnaera Hospital, Busan, Republic of Korea; 3 Department of Orthopedic Surgery, Spine Center, Kangnam Sacred Heart Hospital, Hallym University College of Medicine, 1, Singil-ro, Yeongdeungpo-gu, Seoul, Republic of Korea

**Keywords:** Biportal endoscopic, Open microscopic, Revisional lumbar discectomy, Recurrent lumbar disc herniation, Lumbosacral radiculopathy

## Abstract

**Background:**

Although literature provides evidence regarding the superiority of surgery over conservative treatment in patients with lumbar disc herniation, recurrent lumbar disc herniation (RLDH) was the indication for reoperation in 62% of the cases. The major problem with revisional lumbar discectomy (RLD) is that the epidural scar tissue is not clearly isolated from the boundaries of the dura matter and nerve roots; therefore, unintended durotomy and nerve root injury may occur. The biportal endoscopic (BE) technique is a newly emerging minimally invasive spine surgical modality. However, clinical evidence regarding BE-RLD remains limited. We aimed to compare the clinical outcomes after performing open microscopic (OM)-RLD and BE-RLD to evaluate the feasibility of BE-RLD.

**Methods:**

This retrospective study included 36 patients who were diagnosed with RLDH and underwent OM-RLD and BE-RLD. RLDH is defined as the presence of herniated disc material at the level previously operated upon in patients who have experienced a pain-free phase for more than 6 months. BE-RLD was performed as follows: two independent surgical ports were made inside the medial pedicular line of the target segment and on the intact upper and lower laminas. Peeling off the soft tissue from the vertebral lamina helps to easily identify the traversing nerve root and the recurrent disc material without dealing with the fibrotic scar tissue. Clinical outcomes were obtained using a visual analog scale (VAS) and the modified Macnab criteria before and at 2 days, 2 and 6 weeks, and 3, 6, and 12 months after surgery.

**Results:**

The data of 20 and 16 patients who underwent OM-RLD and BE-RLD, respectively, were evaluated. The demographic and perioperative data were comparable between the groups. During the year following the surgery, in the BE-RLD group, the VAS scores at each point were significantly improved over the baseline and remained improved up to 2 weeks after surgery (*p* < 0.05); however, no statistical difference between the two groups was observed after 6 weeks of surgery (*p* > 0.05). According to the modified Macnab criteria on the follow-up, the excellent or good satisfaction rates reported at 2 weeks, 6 weeks, 6 months, and 12 months after surgery were 81.25%, 81.25%, 75%, and 81.25%, respectively, in the BE-RLD group, and 50%, 75%, 75%, and 80%, respectively, in the OM-RLD group.

**Conclusion:**

BE-RLD yielded similar outcomes to OM-RLD, including pain improvement, functional improvement, and patient satisfaction, at 1 year after surgery. However, faster pain relief, earlier functional recovery, and better patient satisfaction were observed when applying BE-LRD.

**Trial registration:**

Retrospectively registered

## Background

Despite high-quality studies showing that surgical treatment of lumbar disc herniation (LDH) is superior to conservative treatment, reoperation is needed in 15–25% of cases; of these, recurrent lumbar disc herniation (RLDH) was the indication for reoperation in 62% of cases [1–3]. RLDH is defined as the presence of herniated disc material at the level previously operated upon in patients who have experienced a pain-free phase for more than 6 months, and it is considered the primary cause of surgical failure and morbidity in those who have undergone lumbar discectomy [4]. Fortunately, the results of performing revisional lumbar discectomy (RLD) were reported to be favorable [4, 5]. Nevertheless, the peridural fibrotic scar tissue formation inevitably accompanies the primary decompressive laminectomy and is the main reason for difficulties observed when performing open microscopic (OM)-RLD, thus increasing the risk of complications (e.g., unintended durotomy and nerve root injury) and leading to worse clinical outcomes than those observed in primary lumbar discectomy [6, 7].

Biportal endoscopic (BE) technique is a minimally invasive spine surgical modality that is gaining attention worldwide [8, 9]. When BE is applied to symptomatic LDH, it can achieve familiar surgical anatomy from the OM approach with a minimal footprint, regardless of the phase of lumbar degenerative disc disease and the location of disc herniation. Interestingly, good clinical outcomes have also been reported in several studies [10]. Nevertheless, clinical evidence regarding the use of BE-RLD is still lacking.

Therefore, our purpose was to compare the clinical outcomes after performing OM-RLD and BE-RLD to evaluate the feasibility of BE-RLD.

## Methods

Between August 2017 and January 2019, 36 consecutive patients who had lumbosacral radiculopathy with RLDH after undergoing primary lumbar discectomy were enrolled in this study. All participants provided written informed consent. This study was approved by the Institutional Review Board (IRB approval No: 2019-12-022) of Hallym University Medical Center.

The inclusion criteria were the following: (i) previous episode of primary lumbar discectomy, (ii) recurrent lumbosacral radicular pain after a pain-free period following primary lumbar discectomy, (iii) recurrent disc herniation at the same level and direction verified by magnetic resonance imaging (MRI), (iv) refractory axial back and leg radicular pain that had not responded to conservative treatment over 6 weeks, (v) at least 1 year of follow-up, and (vi) age > 18 years. Patients with chronic discogenic pain, but without leg radicular pain, and definite segmental instability combined with spondylolisthesis were excluded.

### Surgical technique

**Fig. 1:**
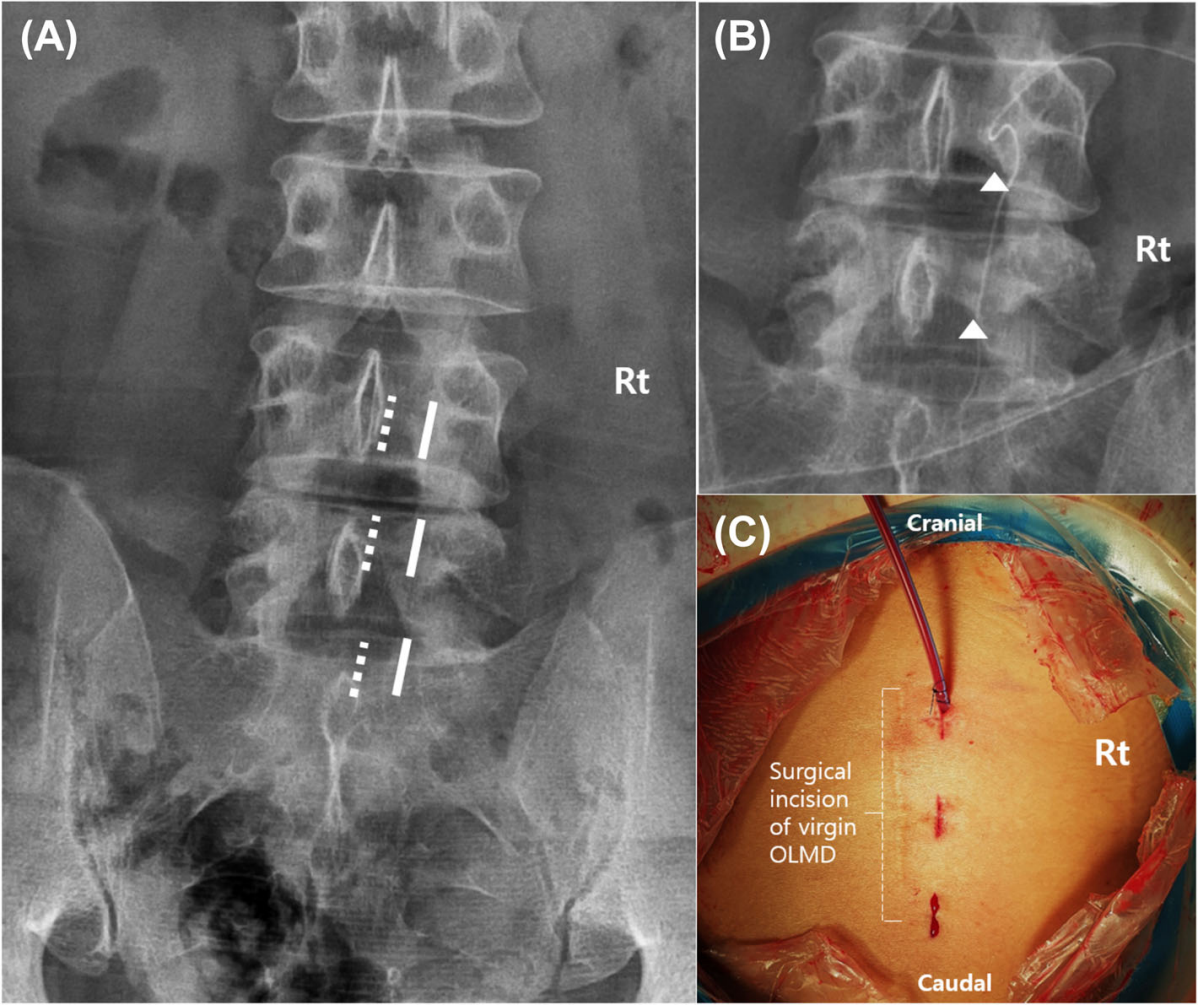
Location of the surgical portal. **a** The viewing and working surgical ports (bold line) were made right inside the medial pedicular line of the target segment and above the intact upper and lower laminas. These were located more lateral than the ports made during conventional biportal endoscopic discectomy (dotted line). **b** Direct access to the lamina and the facet joint was made to complete the red vision discectomy with minimal laminotomy (triangle). **c** Clinical photographs show independent surgical ports on the outside of the previous midline incision site

**Fig. 2:**
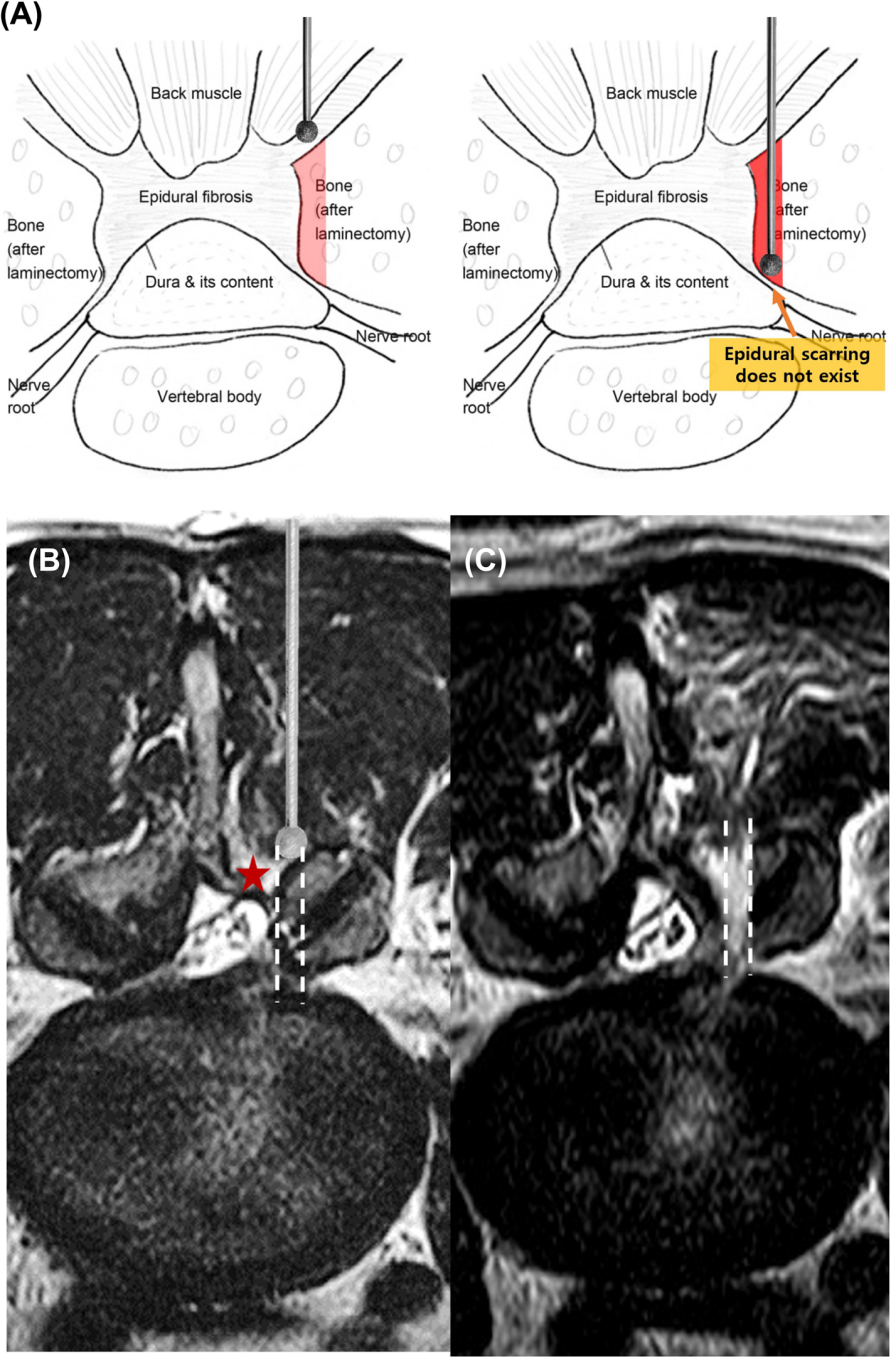
**a** Mimetic diagram of lumbar revision discectomy. **b** The placement of surgical ports laterally with respect to those of the conventional method helps to easily access the epidural space (white bar) without dealing with the fibrotic tissue (red star). **c** Decompressive laminectomy was performed


All patients underwent BE-LRD under general endotracheal or epidural anesthesia. The patients were placed in the prone position on the operating table over a radiolucent Wilson frame in a flexed position. Then, they prepped and draped in a sterile fashion. Under C-arm fluoroscopy, the target level was confirmed and identified on the patient’s skin above the margin of the spinous process, lamina, facet joints, and transverse process. The two independent surgical ports were placed right inside the medial pedicular line of the target segment and on the intact upper and lower laminas (Fig. 1). Placing the surgical ports laterally, rather than using the surgical ports of the BE primary lumbar discectomy, helped to directly access and easily identify the vertebral lamina and inferior articular process without dealing with the fibrotic tissue caused by adhesion with the neural structures in the interlaminar space where the flavectomy was performed [10]. After identifying the laminar and inferior articular processes, more lateral decompression was performed using a diamond high-speed drill and/or chisel, where necessary (Fig. 2). This was followed by a careful release of the traversing nerve root in favor of the adhesion tissue with a small-head (2 mm) angled curette and medial retraction of the traversing nerve root using a dissector to identify the recurrent disc material. Then, annulotomy, radiofrequency annuloplasty, and discectomy were performed meticulously (Video clip 1). The remnant disc fragments under the dura and torn disc space were eliminated with forceful disc irrigation. In cases of observed posterior limbus, circular annuloplasty was performed using a bipolar radiofrequency thermo-controlled ablator (bRFA) to identify and remove the inter-annulus bony fragment (Video clip 2). Complete neural decompression was confirmed by dural pulsation restoration. Bleeding control was achieved using the bRFA and bone wax, and a surgical drain was placed prior to skin closure.

**Additional file 1: Video clip 1**: Annulotomy, radiofrequency annuloplasty, and discectomy were performed meticulously.


**Additional file 2: Video clip 2**: Circular annuloplasty was performed using a bipolar radiofrequency thermo-controlled ablator (bRFA) to identify and remove the inter-annulus bony fragment.

### Postoperative care

**Fig. 3:**
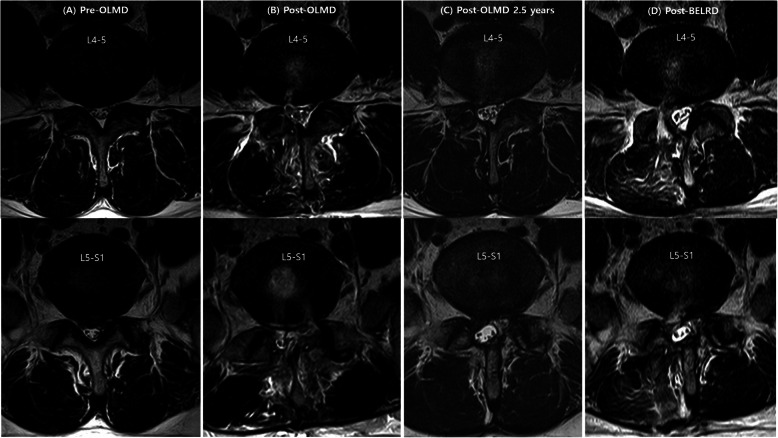
Case presentation: A 39-year-old man with right subarticular recurrent lumbar disc herniation at L4–L5 and L5–S1. **a** Magnetic resonance imaging (MRI) of the right subarticular protruded lumbar disc herniation in the first event. **b** Postoperative MRI after primary open lumbar microdiscectomy. **c** MRI of the recurrent lumbar disc herniation at the same level and direction at 2.5 years after the virgin surgery. **d** Postoperative MRI after biportal endoscopic lumbar redo discectomy: adequate decompression of the sequestrated nucleus and preservation of the facet joint. MRI magnetic resonance imaging

Neurological evaluations were conducted in the recovery room immediate postoperatively. Patients were monitored 24 h after surgery for any complications. Postoperative MRI was performed on postoperative day 1 (Fig. 3).

For postoperative pain control, we employed automatic intravenous patient-controlled analgesia (PCA), using 1 mL of continuous infusion and 1 mL of bolus infusion with a 15-min lockout interval, combined with 25 μg/kg fentanyl, 0.3 mg ramosetron, and saline until postoperative day 2. Additional tramadol injection was used for pain control, as requested by the patients (visual analog scale [VAS] score > 5). After PCA completion, the patients were administered a transdermal 5-mg buprenorphine patch (NORSPAN patch, Mundipharma Korea Ltd., Seoul, Korea) on postoperative week 2.

### Measured data

We recorded demographic and relevant medical history data, including sex, age, body mass index, and the American Society of Anesthesiologists Physical Status Classification System score. Clinical outcomes, including the VAS score (scores of 0 and 10 indicated no pain and the worst pain, respectively) and the modified Macnab criteria, were evaluated. The operation time (skin to skin), length of hospital stay (the duration of hospital stay after operation), amount of surgical drain, and kinetics of serum creatine phosphokinase (CPK) and C-reactive protein (CRP) were recorded. The outcomes were assessed preoperatively, during surgery, and after surgery (i.e., 2 days, 2 weeks, 6 weeks, and at 3, 6, and 12 months). In addition, surgery-related complications were assessed.

### Statistical analysis

Patients were divided into the BE-RLD and OM-RLD groups. All collecting data were compared using the chi-squared test, and the independent *t* test was performed for comparison of continuous variables between the groups. Analyses of perioperative data, modified Macnab criteria, and surgery-related complications were performed using Fisher’s exact test. A *p* value ≤ 0.05 was considered statistically significant. All statistical analyses were performed using SPSS (IBM Corp., Armonk, NY, USA).

## Results

A retrospective review was performed on 36 consecutive patients who underwent BE- and OM-RLD. The demographic and preoperative data were comparable between the two groups (Table 1). The mean pain-free periods after primary lumbar discectomy were 35.75 ± 23.56 (range, 6–85) and 33.55 ± 22.43 (range, 6–86) months in the BE-RLD and OM-RLD groups, respectively; not significantly different (*p* = 0.767).

**Table 1:** Demographic and preoperative data

	Biportal endoscopic revisional lumbar discectomy (***n*** = 16)	Open microscopic revisional lumbar discectomy (***n*** = 20)	***P*** value
**Age**	48.19 ± 8.87	48.80 ± 9.98	0.847
**Sex (male/female)**	9/7	12/8	1.000*
**Body mass index**	24.97 ± 2.79	25.24 ± 2.98	0.691
**ASA score**	1.84 ± 0.36	1.83 ± 0.38	0.779
**Level (%)**			0.451†
**L3–4**	1 (6.25%)	1 (5.0%)	
**L4–5**	10 (62.5%)	10 (50.0%)	
**L5–S1**	5 (31.25%)	9 (45.0%)	
**Direction, right/left (%)**	10/6 (62.5%/37.5%)	12/8 (60.0%/40.0%)	0.883
**Symptom-free period (months)**	33.75 ± 23.56	33.45 ± 22.19	0.767

*ASA* American Society of Anesthesiologists Physical Status Classification System

^*^Chi-squared test

^†^Fisher’s exact test

**Fig. 4:**
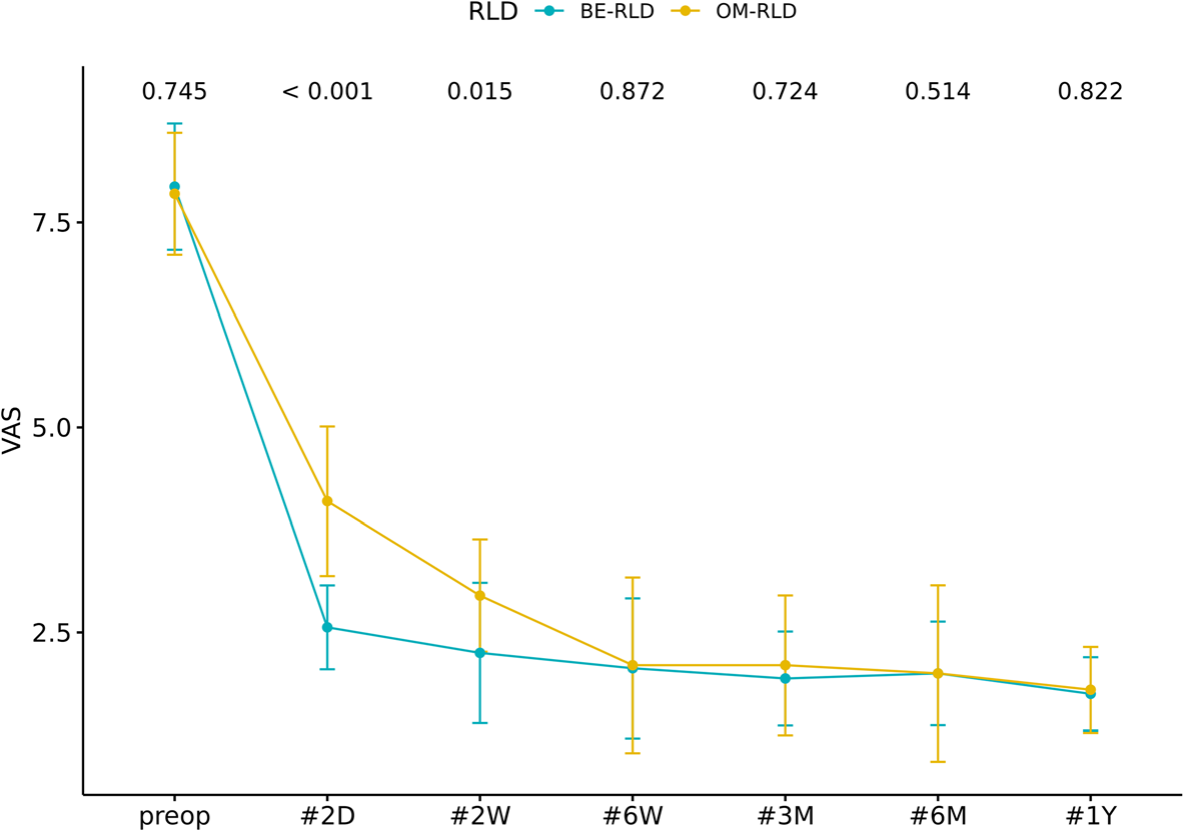
Changes in clinical outcomes between the two surgeries during the 12-month follow-up period

**Fig. 5:**
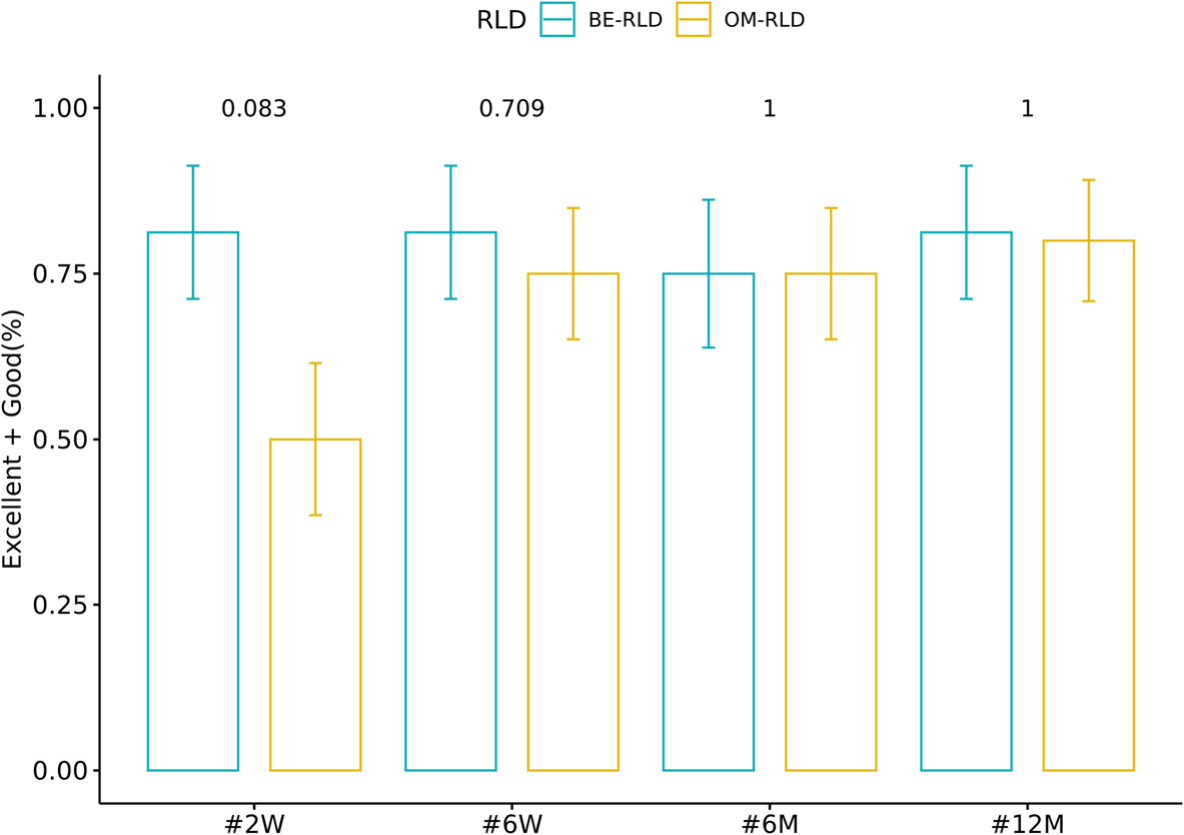
Changes in the ratio of “good” and “excellent,” according to the modified Macnab criteria

In both groups, the VAS scores improved significantly over the baseline value from postoperative day 2 and lasted until the final follow-up examination (*p* < 0.05). However, at 2 days and 2 weeks after surgery, the VAS scores in the BE-RLD group were 2.56 ± 0.51 and 2.25 ± 0.86, respectively, while the corresponding in the OM-RLD group were 4.1 ± 0.91 and 2.95 ± 0.69, respectively (both significantly different: *p* < 0.001 and *p* = 0.013, respectively). No difference in the VAS scores between the two groups from 6 weeks after surgery to the final follow-up was observed (*p* > 0.05) (Fig. 4). According to the modified Macnab criteria on the follow-up, the excellent or good satisfaction rates reported at 2 weeks, 6 weeks, 6 months, and 12 months after surgery were 81.25%, 81.25%, 75%, and 81.25%, respectively, in the BE-RLD group. The corresponding rates in the OM-RLD group were 50%, 75%, 75%, and 80%, respectively. Especially, patient satisfaction at 2 weeks after surgery was better in the BE-RLD group but not statistically significant (*p* = 0.083) (Fig. 5).

The mean operative time and length of hospital stay for the BE-RLD group were 52.81 ± 5.76 min and 2.625 ± 0.72 days, respectively. The corresponding values in the OM-RLD group were 58.00 ± 7.32 min and 4.55 ± 1.96 days, respectively (both significantly different: *p* = 0.023 and *p* < 0.001, respectively). However, the amount of surgical drain was comparable in the two groups (*p* = 0.498). In a perioperative laboratory study, the maximum values of CPK and CRP were 178.34 ± 77.23 IU/L and 2.45 ± 0.46 mg/dL, respectively, in the OM-RLD group, indicating a significant increase from the respective values of 128.52 ± 48.56 IU/L and 0.53 ± 0.39 mg/dL observed in the BE-RLD group (*p* = 0.001). The maximum rise in these levels was observed on postoperative day 1 in both groups. The serum CRP levels recovered to the normal range on postoperative day 2 in the BE-RLD group and on 1 week after surgery in the OM-RLD group. Moreover, the serum CPK levels recovered to normal range on postoperative days 2 and 3 in the BE-RLD and OM-RLD groups, respectively (Table 2).

**Table 2:** Operative data, laboratory outcomes, and complications

	Biportal endoscopic revisional lumbar discectomy (***n*** = 16)	Open microscopic revisional lumbar discectomy (***n*** = 20)	***P*** value
**Total operation time (min)**	52.81 ± 5.76	58.00 ± 7.33	**0.023**
**Amount of surgical drain (mL)**	66.25 ± 20.62	59.50 ± 37.52	0.498
**Length of hospital stay (days)**	2.62 ± 0.72	4.55 ± 1.96	**< 0.001**
**CRP (mg/dL)**			
**Preoperative**	0.12 ± 0.27	0.11 ± 0.20	0.963
**Postoperative day 1**	0.53 ± 0.39	2.45 ± 0.42	**0.001**
**Postoperative day 2**	0.23 ± 0.44	1.09 ± 0.72	**0.03**
**Postoperative day 7**	0.17 ± 0.27	0.20 ± 0.46	0.29
**CPK (IU/L)**			
**Preoperative**	106.21 ± 50.75	102.35 ± 57.20	0.927
**Postoperative day 1**	128.52 ± 48.56	178.34 ± 77.23	**0.014**
**Postoperative day 2**	103.38 ± 46.62	113.52 ± 51.20	0.168
**Complications**			1.000†
**Incidental durotomy**	1 (6.3%)	2 (10%)	
**Epidual hematoma**	0 (0%)	1 (5%)	
**Local recurrence**	2 (12.5%)	3 (15%)	

*CPK* creatine phosphokinase, *CRP* C-reactive protein

^†^Fisher’s exact test

One case of incidental durotomy and two cases of persisted leg dysthesia occurred in the BE-RLD group. Two, one, one, and two cases of incidental durotomy, epidural hematoma, disc local recurrence, and persisted leg dysthesia, respectively, occurred in the OM-RLD group. No patient required revision surgery for sustained or aggravated symptoms during the entire follow-up period.

## Discussion

The main findings of this retrospective study were the following: (1) BE-RLD and OM-RLD showed good clinical outcomes at 1 year after surgery, (2) BE-RLD performance resulted in significant improvement in pain and patient satisfaction up to 2 weeks after surgery, and (3) the peak serum CRP and CPK values were significantly higher in the OM-RLD group and took longer to recover to the normal range.

Postoperative epidural fibrosis (PEF), which corresponds to the development of a dense scar tissue adjacent to the dura matter after decompressive laminectomy, is in fact a physiologic process of wound healing [11]. Fibroblasts that generate PEF are derived from the adjacent paraspinal musculature. This physiologic scar tissue may become an extradural hypertrophic enveloping film, the so-called post-laminectomy membrane. This extradural fibrotic membrane may extend into the vertebral canal and adhere to the dura matter and nerve roots, often causing recurrent symptoms [12, 13]. PEF tends to form a curvilinear pattern surrounding the dural sac following the contour of the inner laminar surface [14]. The most important issue with RLD is that PEF is not clearly isolated from the boundaries of the dura matter and nerve roots; therefore, unintended durotomy and nerve injury may occur, and segmental instability may be caused by excessive removal of the posterior structures, including the facet joint [15].

The recently introduced BE for spine surgery may restore familiar surgical anatomy that can be accessed via the conventional approach with only a minimal footprint. This modality requires two independent working and viewing ports, through which, continuous fluid irrigation is performed in the workspace where an independent space within an epidural space is made by peeling the multifidus from the vertebral lamina [16]. The use of these irrigation fluids may help to maintain the field of view and to secure the workspace. Furthermore, the use of normal saline, as a medium, could reduce thermal nerve root injury when drilling the bone around the nerve tissue with the high-speed diamond drill during laminotomy [17]. Another merit of performing a surgery under continuous fluid irrigation is that it allows the use of an advanced energy-based surgical dissection device, called bRFA. The latter causes less thermal injury, less lateral tissue damage, better vascular coagulation, and better tissue healing compared to electrocautery [18–20]. bRFA helps to achieve effective hemostasis without the possibility of causing electrical and thermal damage to the nerve tissue when the operator is uncertain of the nerve location while controlling bleeding in the peridural scar tissue.

In biportal endoscopic primary lumbar discectomy, the surgical ports are placed at the lateral boundary of the given spinous process, and the authors suggested that, in revision surgery, the surgical ports should be placed more laterally and above the medial interpedicular line [10]. As laminotomy and flavectomy have already been performed in the primary surgery, this setup of surgical ports helps to allow direct approach to the intact vertebral lamina and inferior articular process, making it easier to access the recurrent disc material, while touching the fibrotic scar tissue less and minimizing additional laminotomy and facetotomy (Fig. 2). Moreover, to identify the proper surgical anatomy in RLD, more surgical dissection is often required compared to the primary surgery. Additionally, as biprotal endoscopic technique provides real-time magnification images of the surgical field through the endoscope, only a limited surgical dissection can provide sufficient surgical field to perform meticulous lumbar discectomy. Although no significant differences were observed in this study, this may be the reason that the total operation time was lower in the OM-RLD than in the BE-RLD group. In addition, this may reduce the potential risk of delayed spondylolisthesis and consequent segmental instability [10].

In previous works, muscle damage and systemic inflammatory responses that inevitably occur during surgery were evaluated by estimating the serum CPK and CRP levels in a laboratory test, respectively, and it was reported that endoscopic primary lumbar discectomy causes less muscle damage and systemic inflammatory response than microscopic primary lumbar discectomy [21]. In this study, the laboratory results of RLD showed that BE also causes less muscle damage and systemic inflammatory response compared to OM. Moreover, we considered that these results were attributed to continuous fluid irrigation and low thermal damage caused by the use of bRFA. Especially, BE spinal surgery is considered to be a thermo-controlled surgery, with substantial advantages for revisional spine surgery, including the absence of the ligamentum flavum around the nerve root and thecal sac, similar to those of the primary surgery.

In RLDH, conservative treatment is not feasible, and additional surgery must be performed. The results of performing OM-RLD were reported to be favorable, but inferior to the primary microscopic lumbar discectomy [5]. For this retrospective study, favorable clinical outcomes were obtained at 1 year after performing BE-RLD and OM-RLD. In particular, our results were similar to those of a previous work that performed BE primary lumbar discectomy, including the short operating time, low estimated bleeding loss, short length of hospital stay, immediate reduction of axial back and leg pain after surgery, and high patient satisfaction [9, 10]. There have been no cases of lumbar arthrodesis surgery because of the segmental instability caused by excessive resection of the facet joint.

This study had certain limitations. First, the sample size was small, and there was a relatively short-term follow-up period. In addition, there was heterogeneity in the type and length of conservative treatment, conducted in all patients before and after surgery. However, our study had the strength of providing the technical update of biprotal endoscopic spine surgery and presented the clinical outcomes. Therefore, prospective randomized controlled studies should be conducted including a larger sample size to divide the participants into groups and make the appropriate comparisons.

## Conclusion

Our study suggested that BE-RLD is an alternative surgery option and presents similar clinical outcomes as OM-RLD at 1 year after surgery. However, faster pain relief and earlier functional recovery were observed in BE-LRD.

## Data Availability

All data generated or analyzed during this study are included in this published article.

## Abbreviations

RLDH: Recurrent lumbar disc herniation
BE: Biportal endoscopic technique
BE-RLD: Biportal endoscopic revisional lumbar discectomy
bRFA: Bipolar radiofrequency thermo-controlled ablator
MRI: Magnetic resonance imaging
OM-RLD: Open microscopic lumbar discectomy
PCA: Patient-controlled analgesia
PEF: Postoperative epidural fibrosis
VAS: Visual analog scale
